# Comparison of the Effects of Goat Dairy and Cow Dairy Based Breakfasts on Satiety, Appetite Hormones, and Metabolic Profile

**DOI:** 10.3390/nu9080877

**Published:** 2017-08-15

**Authors:** Elehazara Rubio-Martín, Eva García-Escobar, Maria-Soledad Ruiz de Adana, Fuensanta Lima-Rubio, Laura Peláez, Angel-María Caracuel, Francisco-Javier Bermúdez-Silva, Federico Soriguer, Gemma Rojo-Martínez, Gabriel Olveira

**Affiliations:** 1 UGC Endocrinología y Nutrición, Instituto de Investigación Biomédica de Málaga (IBIMA), Hospital Universitario Regional de Málaga/Universidad de Málaga, 29009 Malaga, Spain; elehazara@gmail.com (E.R.-M.); solruizdeadana@gmail.com (M.-S.R.d.A.); santi.lima@hotmail.com (F.L.-R.); laupelaez_79@hotmail.com (L.P.); angelm.caracuel.sspa@juntadeandalucia.es (A.-M.C.); javier.bermudez@ibima.eu (F.-J.B.-S.); federicosoriguer@gmail.com (F.S.); gemma.rojo.m@gmail.com (G.R.-M.); gabrielm.olveira.sspa@juntadeandalucia.es (G.O.); 2 Centro de Investigación Biomédica en Red (CIBERDEM CB07/08/0019), Instituto de Salud Carlos III, 29009 Malaga, Spain

**Keywords:** goat dairy, cow dairy, satiety, appetite regulation, body weight control, metabolic profile

## Abstract

The satiating effects of cow dairy have been thoroughly investigated; however, the effects of goat dairy on appetite have not been reported so far. Our study investigates the satiating effect of two breakfasts based on goat or cow dairy and their association with appetite related hormones and metabolic profile. Healthy adults consumed two breakfasts based on goat (G-Breakfast) or cow (C-Breakfast) dairy products. Blood samples were taken and VAS tests were performed at different time points. Blood metabolites were measured and Combined Satiety Index (CSI) and areas under the curves (AUC) were calculated. Desire to eat rating was significantly lower (breakfast & time interaction *p* < 0.01) and hunger rating tended to be lower (breakfast & time interaction *p* = 0.06) after the G-breakfast. None of the blood parameters studied were different between breakfasts; however, AUC_GLP-1_ was inversely associated with the AUC_hunger_ and AUC_desire-to-eat_ after the G-Breakfast, whereas triglyceride levels were directly associated with AUC_CSI_ after the C-Breakfast. Our results suggest a slightly higher satiating effect of goat dairy when compared to cow dairy products, and pointed to a potential association of GLP-1 and triglyceride levels with the mechanisms by which dairy products might affect satiety after the G-Breakfast and C-Breakfast, respectively.

## 1. Introduction

It is well established that Mediterranean diet has potential beneficial effects on the treatment of Metabolic Syndrome and related comorbidities such as Type-2 Diabetes, cardiovascular diseases and obesity [1]. The traditional Mediterranean diet is characterized by a high intake of fruits and vegetables, whole grains, legumes, nuts, fish and olive oil but it also includes moderate consumption of goat milk and dairy products [2].

Although it may not be important in certain parts of the world, goat milk plays an important role in the economic development and diet in the areas called “the cradle of modern civilization” as well as in many developing countries, including Mediterranean, Middle East, Eastern Europe and South American countries; in fact worldwide goat milk production has increased by 44.10% from 2000 to 2014, from 12,726,469 to 18,340,016 metric tons.

In developed countries, goat milk production is becoming economically relevant, mainly due to the production of goat’s cheeses [3,4]. Another reason for the demand of goat milk derives from the increasing incidence of cow’s milk allergy and other gastrointestinal diseases. Cow’s milk allergy is the most frequent allergy in the first years of life, so milk from other mammalian species, including goat, has been suggested as possible alternatives [5]. 

Consumption of goat milk and dairy products has also been associated with beneficial health effects [6,7,8]. Accordingly, several studies have proposed that anti-oxidant and anti-inflammatory effects of goat milk are associated to its particular fatty acid composition [7,9], as well as to its immunomodulatory and anti-atherogenic properties [8]. 

There are many qualitative differences between goat milk and milk produced by other domestic species [3]. Goat milk has different amounts of some vitamins, minerals and proteins when compared to cow or sheep milk [3,10], which could be related to better digestibility and benefits in some diseases such as malabsorption syndrome [3,11]. Fatty acid composition of goat milk is also different from that of cow milk, containing higher amount of medium-chain FAs (caprylic acid (C8) and, more markedly, capric acid (C10)), while cow milk is higher in butyric (C4) and, sometimes, palmitic (C16:0) acids [7]. Medium-chain FAs have a high nutritional relevance [12] because they are rapidly oxidized by the liver, inducing a fast satiating effect [13,14].

Obesity and overweight are considered to be a general health problem due to their association with a high number of metabolic diseases, including insulin resistance, type 2 diabetes mellitus and cardiovascular diseases. Both obesity and overweight are the result of a positive energy balance due to energy intake exceeding energy expenditure. The most employed strategy to lose weight is caloric restriction, which in most cases leads to poor results at the long-term [15], so consumption of more satiating macronutrients could be a good alternative approach.

In order to unravel new factors affecting hunger and satiety, researchers have begun studying specific nutrients or bioactive food compounds in peoples’ diets that may impact these processes. The satiating effect of dietary protein has been previously studied [16,17] with a general conclusion being that relatively high-protein diets could be an effective tool for body weight loss and weight maintenance after weight loss. Although early studies have shown weaker satiating effects of dietary fat when compared to isoenergetic amounts of protein [18,19], dietary fats have also been described as important regulators of satiety signals [13,20]. 

Due to the high protein content of cow milk and its unique fatty acid composition, its satiating effects has been investigated, showing that consumption of cow dairy products can increase satiety [21,22,23,24]. However, despite the increasing production and interest regarding goat milk over the last years [3,4], including the possible greater benefits of goat dairy over cow dairy [3,7,8], to the best of our knowledge there are no studies comparing the satiating effects of goat dairy, one of the traditional component of the Mediterranean diet [2], to those of cow dairy. With this in mind, the aim of our work was to investigate the satiating effects induced by two isocaloric breakfasts based on these two milks: goat dairy breakfast (G-Breakfast) versus cow dairy breakfast (C-Breakfast), and their association with appetite related hormones (ghrelin and GLP-1), insulin, circulating lipids and glucose levels.

## 2. Materials and Methods

### 2.1. Participants

The participants in this study were recruited from the staff of Malaga’s Hospital. They were contacted by email and asked for participation in this study. A total of 33 healthy volunteers (18–65 years) were included in the study. None of the participants were accustomed to consuming goat milk. Each participant underwent a physical examination: height, weight, waist and hip circumference were measured and Body Mass Index (BMI) was calculated as weight/height^2^. A written informed consent was obtained from all participants and the study protocol was approved by the Ethics and Clinical Investigation Committee of Hospital Regional de Malaga. 

### 2.2. Study Protocol

The study was an open randomized cross-over trial. Randomization was performed by using a random number table. The study consisted of two randomized experimental breakfasts separated by at least 7 days of washout period. All subjects had a test breakfast based on goat dairy (G-Breakfast) as well as a control breakfast based on cow dairy (C-Breakfast).

The protocol started at 08:30 h after an overnight fast, which started at 22:00 h. Each test day a catheter was placed in an antecubital vein to collect the blood samples. A basal blood sample was taken and appetite ratings were scored (Time 0). Then breakfast was served and completed within 10 min. Breakfast consisted of 200 mL of commercial UHT semi-skimmed goat or cow milk, supplied by Covap (Cordoba, Spain), 40 g of white bread and 40 g of commercial goat semi-cured cheese supplied by Corsevilla (Sevilla, Spain), or 40 g of cow semi-cured cheese (Quesos Cerrato Soc. Coop., Palencia, Spain) bought in a supermarket. Both cheeses were produced from pasteurized milk, followed by a similar ripening process. Macronutrient composition and energy content of each cheese and milk as well as that of the whole breakfast are shown in Table 1. Basically, the two breakfasts were quite similar. As the amount of bread consumed in both breakfast was the same, the possible limitation due to the interference of the bread in the satiating effects was controlled. Eating or drinking any other food or beverage during the test was not allowed. During both breakfasts the room temperature was maintained between 18 and 20 °C and 48 h before the second visit all the participants were asked to mimic the first visit as closely as possible.

Appetite ratings were completed just before breakfast (Time 0) and at 30′, 60′, 90′, 120′, 180′ after breakfast. A final test was also conducted just before lunch (14:00 hours) to evaluate what level of satiety the subject had at lunchtime depending on the type of breakfast ingested. Blood samples were taken at the following time points: 15′, 30′, 60′, 90′, 120′ and 180′, and immediately processed according to routine procedures. They were stored at −80 °C, for later analysis, in the Biobank of Málaga’s Hospital, which belongs to the regional Biobank of the Andalusian Public Health System (project PT13/0010/0006).

### 2.3. Appetite Profile

To determine the subjective appetite profile, hunger, fullness, satiety, and desire to eat were rated on 10 cm visual analogue scales (VAS), anchored with ‘not at all’ and ‘extremely’ during the test days. VAS is often used to measure subjective appetite sensations and their validity and reproducibility have been shown in previous studies [23]. Subjects were instructed to rate themselves by marking the scale at the point that was most appropriate to their feeling at that time. 

With data derived from the VAS test, Combined Satiety Indexes (CSI) [24] was calculated by the following formula: CSI = [Fullness + (10-desire to eat) + (10-Hunger) + (10-Prospective food consumption)]/4. CSI values are within the range of 0 to 10, 0 being the maximum appetite sensation and 10 the minimum appetite sensation. This index can give us an overall measure of satiety [24]. Areas under the curves (AUC) of the test response and all other determinations were also calculated.

### 2.4. Blood Parameters

Circulating glucose, free fatty acid (FFA), total cholesterol, HDL-cholesterol and triglyceride levels were determined by enzymatic techniques in an A15 auto-analyzer from Biosystems S.A. (Barcelona, Spain). 

Plasma concentrations of the acylated ghrelin and GLP-1 were quantified by specific ELISA kits (SPIBIO, BertinPharma, Montigny-le-Bretonneux, France and Phoenix Pharmaceuticals, INC, Karlsruhe, Germany, respectively). Insulin levels were determined by RIA (Coat a Count RIA kit, DPC, Los Angeles, CA, USA).

### 2.5. Statistical Analysis

Due to the fact that no previous studies have analyzed the effects of goat dairy on appetite related hormone levels, the sample size (*n* = 33) was based on a previous research by Veldhorst et al. [14] that investigated the influence of different breakfasts containing casein, whey or soy as protein sources on satiety and appetite hormones in healthy subjects. According to this report, a study able to detect changes over 10% in the circulating appetite related hormones, with an alpha = 0.05, needs a minimum sample size of 32 subjects.

Data are presented as mean with their standard errors, unless otherwise indicated. Statistical significance was assumed at *p* < 0.05, unless otherwise stated.

The area under the curve (AUC) for plasma levels of appetite related hormones and the subjective feeling of appetite were calculated from before breakfast to 180 min after breakfast, for metabolite plasma levels, and at lunchtime for the appetite ratings, using the trapezoidal rule (GraphPad Prism software, San Diego, CA, USA). Our AUC calculation included all areas above and below baseline. 

The effect of time and the possible differences between test and control breakfasts on fasting and postprandial levels of metabolites/hormones and subjective feelings of appetite were analyzed by a repeated measure unadjusted ANOVA or ANCOVA adjusted by the significantly associated variables in lineal regression models. Where a significant effect of time or breakfast appears, the paired *t*-Student test corrected by Bonferroni test for multiple comparisons was employed. Differences in AUCs were evaluated by paired sample *t*-Student test. Pearson (or Spearman) correlations were used to test the relationship between changes in the plasma levels of the hormones measured, changes in subjective feelings of appetite and other anthropometric variables.

## 3. Results

### 3.1. Baseline Characteristics

A total of 33 subjects (16 females and 17 males, aged 35.6 ± 9.4 years, body mass index: 26.29 ± 6.19 kg/m^2^, body weight: 71.18 ± 22.30 kg, waist to hips ratio: 0.84 ± 0.07, male waist circumference: 87.74 ± 26.73 cm, female waist circumference: 83.06 ± 10.70 cm) completed both experimental breakfasts with no differences in their basal characteristics between the test days (Table 2).

### 3.2. Appetite Ratings (VAS)

**Figure 1:**
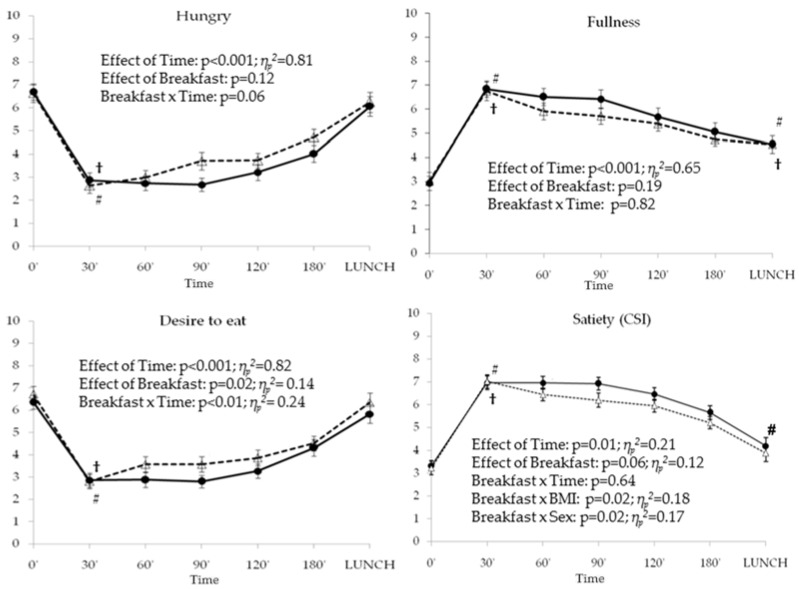
Appetite Ratings (Visual Analog Scale). Average and standard errors for VAS score (cm) after G-Breakfast (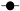) and C-Breakfast (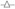) at the different time points. The effects of time, breakfast and interactions were calculated by repeated measures ANOVA, except for CSI index that were calculated by repeated measures ANCOVA adjusted by Sex and BMI. # Statistically different means compared to baseline (0′) after G-Breakfast. † Statistically different compared to baseline (0′) after C-Breakfast.

Changes in the appetite ratings were different between breakfasts only in the desire to eat rating with an effect size estimated of 14.5%. Changes in prospective food intake were similar to the changes in hungry rating and are therefore not presented separately. In our study, G-Breakfast induced lower desire to eat in the subjects than the C-Breakfast (Figure 1). The effects of breakfasts on CSI rating were close to be significant (*p* = 0.056) (Figure 1). Additionally, significant interactions arising between Breakfast and BMI and Breakfast and sex associated to the CSI rating variability (Figure 1).

In Figure 1 we can also observe a significant main effect of time (*p* < 0.001) for all appetite ratings with an interaction with the breakfast only for the desire to eat rating that explains 24% of its variability. For both breakfasts, ratings of hunger and desire to eat were significantly lower immediately post-breakfast (T30′) (*p* < 0.001 for both ratings) (Figure 1) with an increment in these ratings from that moment until lunch time when they were no different than before breakfast (T0′). Ratings of satiety and fullness were significantly higher after breakfast (*p* < 0.001 for both breakfasts, Figure 1) compared to baseline, and were subsequently decreasing until lunchtime. Fullness ratings at lunchtime were higher than before breakfast for both breakfasts (*p* < 0.01 for G-Breakfast, *p* < 0.001 for C-Breakfast. Figure 1); however, the satiety index at lunchtime was significantly higher than before breakfast only for the G-Breakfast (*p* = 0.02, Figure 1).

The AUC for the appetite ratings were not different between breakfasts, however differences in the AUC for the combined satiety index between breakfasts were close to being significant (AUC_CSI_ for G-Breakfast: 953.69 ± 36.52; AUC_CSI_ for C-Breakfast: 894.77 ± 37.27; *p* = 0.06).

### 3.3. Appetite-Related Hormones

**Figure 2:**
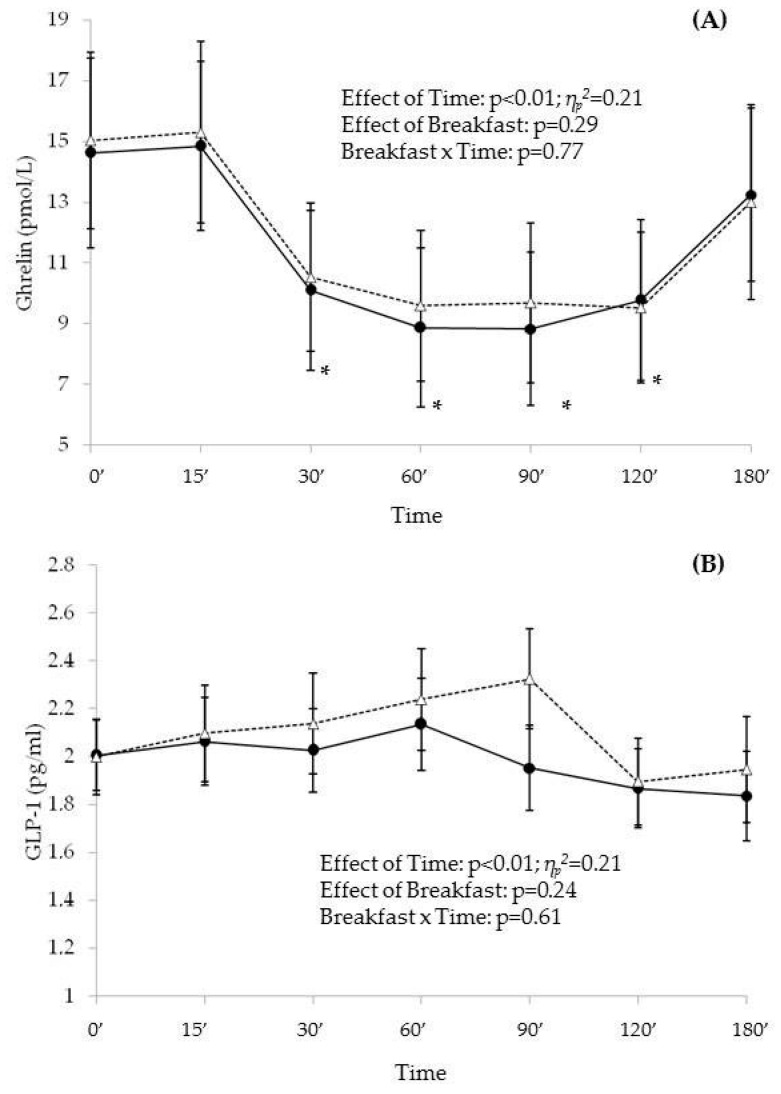
Appetite hormones responses. Average and standard error for the appetite-related hormones ghreline (**A**) and GLP-1 (**B**) after G-Breakfast (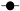) and C-Breakfast (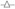) at the different time points. The effects of time, breakfast and interactions were evaluated by repeated measures ANOVA. * Statistically different means versus baseline (0′) after G-Breakfast and C-Breakfast.

There were no differences between the two breakfasts for plasma acylated ghrelin concentrations nor for the AUC of this hormone; however, a significant main effect of time for the acylated ghrelin levels was observed without interacting with the breakfast (Figure 2). 30 min after both breakfasts, acylated ghrelin was significantly lower compared to T-0′ (*p* = 0.003 for G-Breakfast, *p* < 0.001 for C-Breakfast. Figure 2) and the levels remained lower at T-120′. Ghrelin levels at T-180′ were increased when compared to T-120′, recovering fasting levels. AUC for Ghrelin levels was negatively associated with CSI at lunchtime for both breakfasts (*r* = −0.44, *p* = 0.01 for G-Breakfast; *r* = −0.47, *p* = 0.01 for C-Breakfast).

Similar to ghrelin levels, GLP-1 levels were not different between breakfasts, but a significant effect of time was found with no interaction between time and breakfast (Figure 2). There were no differences in the AUC for this hormone between the breakfasts.

AUC for GLP-1 was inversely associated with the AUCs for the appetite ratings hunger and desire to eat only after the G-Breakfast (*r* = −0.37, *p* = 0.04 and *r* = −0.38, *p* = 0.03, respectively).

### 3.4. Plasma Metabolites and Insulin

**Figure 3:**
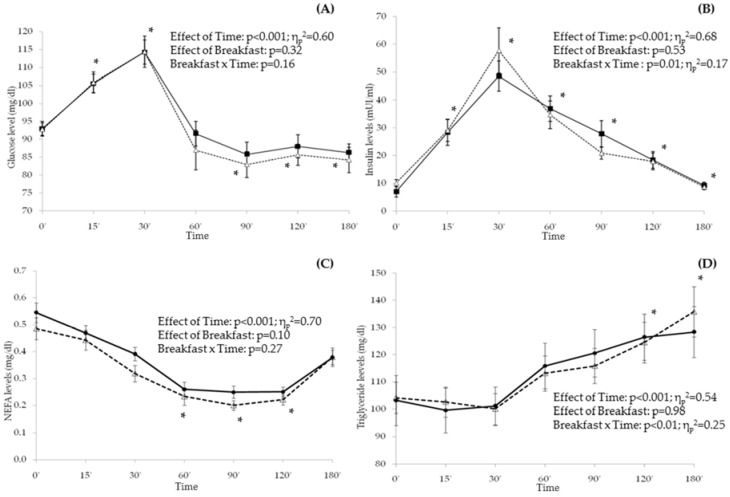
Metabolic profile. Average and standard error for circulating glucose (**A**), Insulin (**B**), NEFA (**C**) and triglycerides (**D**) levels after G-Breakfast (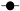) and C-Breakfast (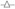) at the different time points. The effects of time, breakfast and interactions were evaluated by repeated measures ANOVA. * Statistically different means versus baseline (0′) after G-Breakfast and C-Breakfast.

A significant main effect of time, but no effect of breakfast or interaction, was observed on glucose, insulin, triglyceride and FFA plasma levels (*p* < 0.001 for all of them) (Figure 3). Both glucose and insulin plasma levels increased from breakfast until T-30′ and decreased afterward. 

For both breakfasts, glucose levels from T-90′ to T-180′ were significantly lower than in fasting condition, while insulin levels remained significantly higher than before breakfast by T-120′ (Figure 3). There was no association between AUCs for any of the appetite ratings and insulin or glucose levels.

NEFA levels gradually decreased from breakfast until T-120′, with the lowest values being between T-60′ and T-120′; on the contrary, triglyceride levels remained unchanged until T-30′ and then they continuously increased until T-180′. The AUC for the triglycerides levels were directly associated with AUC for CSI, but only in the C-Breakfast (AUC_TG_–AUC_CSI_: *r* = 0.37, *p* = 0.03). 

A main effect of time, with no interaction with breakfast, was found in circulating levels of cholesterol and HDL-cholesterol (data not shown).

## 4. Discussion

To the best of our knowledge, this is the first study to evaluate the impact of goat milk and goat dairy on appetite ratings in adults. Our primary aim was to compare the effect of a goat dairy-based breakfast on the subjective appetite response, measured by the VAS scale, with a cow dairy-based breakfast. Overall, the results obtained from the appetite rating analysis indicated that although time was the main factor influencing the subjective appetite response, desire to eat showed a differential response after the two breakfasts, the response being lower after the G-Breakfast. It has been reported that studies with effect sizes that are small but nevertheless significant because of large sample sizes can overestimate the observed effect [25]; however, considering our sample size (33 subjects), we believe that the effects sizes of the type of breakfast found on subjective appetite response in our study were not overestimated.

It is well known that food macronutrient composition (the relative amounts of protein, fat and carbohydrate) exhibits a direct effect on short-term food intake suppression and satiety [13]. Proteins, generally agreed to be the most satiating macronutrient, differ in their effects on appetite depending on their composition, absorption and digestion [13,18]. Dietary fats are also a strong regulator of satiety signals, mainly through their influence on gut hormones release [26]. And finally, dietary carbohydrates affect appetite feelings in different ways, for example sugars and starches influence satiety and short-term food intake primarily through their effect on blood glucose and insulin responses [27], while the effect of dietary fibers more likely occurs via modulation of gastric motor function and blunting of postprandial glucose and insulin responses [28]. But for all of them, the macronutrient dose and source are considered to be important determinants of food intake regulation [13]. In our study, the meal macronutrient composition and energy content was almost the same for both breakfasts, so the macronutrient dose should not be interfering the satiety effect of each meal. Even though the G-Breakfast had a slightly lower energy content, total protein and fat composition than C-Breakfast, which could be expected to induce a slightly higher satiety effect after the C-Breakfast, our results showed that, in contrast, G-Breakfast was significantly associated to reduced desire to eat rating and was almost significantly associated to increased CSI index and AUC_CSI_ compared to C-Breakfast. Our results also showed that women or subjects with high BMI were more satiated after the G-Breakfast than after the C-Breakfast (data not shown). Additionally, we found that the CSI index was significantly higher at lunchtime than before breakfast only after the G-Breakfast. Together, all these findings support the importance of the macronutrient source as determinant of their satiety effects and points to higher satiating effects of goat dairy when compared to cow dairy products.

A recent meta-analysis assessing the effects of dairy product consumption on satiety and food intake [21] has reported that the effects of consumption of dairy products on subjective satiety indicators depend on the volume of dairy products ingested, more than 500 mL of dairy products being necessary to significantly increase satiety indicators. According to this, the small amount of dairy products consumed by our volunteers could be considered a limitation of our study, and might be related to the moderate effect on satiety feeling observed after the G-Breakfast when compared with the C-Breakfast. 

It is well accepted the reliability and validity of VAS in terms of their ability to measure subjective appetite feelings, their sensitivity to experimental manipulations, and their reproducibility; however, VAS correlates with, but do not reliably predict, energy intake [23]. Inconsistent associations between VAS scores after intake of different nutrients and subsequent energy intake at lunch or during the day have been reported. Several studies showed a direct relationship between satiety scores after breakfasts with different fatty acid chain lengths [29], protein percentages [30] or bran fiber contents [31] and food intake at a subsequent meal [29,30,31] or over 24 h [31]; however, other authors reported no association between satiety ratings and ad libitum energy intake at lunch when comparing breakfasts with different percentages of proteins from different sources (casein, soy or whey) [16,32,33,34]. In line with these results, Pal et al have reported that the short-term effects on satiety from dairy whey proteins did not have any long-term effects on energy intake or body weight over 12 weeks compared with casein in overweight and obese individuals [35]. Because of these inconsistent results, it is not possible to predict whether the differences in VAS scores after the goat dairy and cow dairy in our study will translate into short-term or longer-term differences in energy intake and body weight. This has to be investigated in future studies.

Our second aim was to determine the impact of each breakfast on metabolic parameters (appetite-related hormones, glucose, insulin and blood lipid profile) that have been previously suggested as mechanisms by which dairy products could be affecting appetite [21,36] and their association with the subjective feelings of satiety. It has been proposed that several components of dairy like protein, fat or calcium, may have a role in appetite regulation by modulating appetite hormones such as ghrelin and GLP-1 [17,23,24]. In our study, acylated ghrelin and GLP-1 plasma levels were no different between breakfasts, however AUC for the anorexigenic hormone GLP-1 was inversely correlated with the AUC for the appetite ratings “hunger” and “desire to eat” only after the G-Breakfast, so it is tempting to speculate a possible involvement of GLP-1in the satiating effects of goat dairy, though more research is needed.

It is unclear whether postprandial blood glucose exerts a regulatory function in short-term appetite regulation in humans. The glucostatic theory proposes that increments in blood glucose concentrations result in increased feelings of satiety, whereas a drop in blood glucose concentrations has the opposite effect [27]; however, other investigations have concluded that these postprandial glucose levels are not associated with appetite regulation in healthy subjects [37]. Dairy products have been proposed to reduce the dietary glycemic index, which might regulate appetite [38]; however, later investigations have revealed that this index is not useful for predicting their effects on satiety or food intake within mixed meals [39]. Supporting the previous findings disassociating post-meal circulating glucose levels and appetite feelings in healthy subjects [37], our study showed no differences between breakfasts in glucose levels, with no association between glucose levels and appetite ratings. 

Although the protein content of dairy has been postulated to be involved in increased plasma insulin levels due to their insulinotropic properties [40], there is some controversy about whether this could affect appetite. In the same way that some authors have found a strong relationship between self-rated appetite, postprandial insulin response and energy intake at lunch [10,41], others have proposed that post-meal variations in insulinemia have inconsistent effects on subjective appetite ratings [42,43]. In our study the consumption of a goat or cow dairy based breakfast did not lead to differences in plasma insulin levels. Moreover, no association between insulin levels and the appetite ratings were found in our study supporting previous studies reporting no association between postprandial insulinemia and subjective appetite. 

Regarding the blood lipid profile, recent studies have highlighted the importance of changes in circulating lipids, particularly triglycerides, as a strong predictor for hyperphagia and obesity [44,45]. Indeed, increased circulating lipids in the blood could distort serotonergic signaling, thus altering satiety and hunger signals [36]. Together, these observations raise the possibility that nutritional lipids, particularly triglycerides, directly affect cognitive and rewarding circuits, contributing to reduce subjective appetite at the central level in the non-obese condition [46]. Our results have shown that neither triglyceride, FFA, HDL-cholesterol or cholesterol levels were different between breakfasts. These results are consistent with previous articles reporting similar postprandial plasma triglyceride, cholesterol or FFA after three test meals based on different dairy products [47] or after the supplementation of four different whey protein fractions to a fat-rich meal [48]. However, a direct association between AUC for triglyceride levels and the AUC for the combined satiety index was found after the C-Breakfast but not after G-Breakfast in our study. This difference between breakfasts might be explained by the combination of the different FA composition and triglyceride structure (i.e., high proportion of C6-C10 FA esterified at carbon [3] of goat milk compared to cow milk [3,49]. As it is known, goat milk contains higher amount of medium-chain fatty acids in their triglycerides, while cow milk contains more long-chain fatty acids. In contrast to chylomicrons containing long-chain triglycerides, those containing medium-chain triglycerides, which indeed contain saturated FAs with carbon chain lengths of 6–10 atoms, are mostly absorbed into the portal system and are rapidly oxidized by the liver [13], so it is possible that triglycerides from goat dairy may not be allocated to be hydrolyzed by the brain lipoprotein lipase with the subsequent appetite regulation effect [44], while the long-chain triglycerides present in cow milk fat may be acting on satiety feelings through this central mechanism [46].

## 5. Conclusions

In a context in which metabolic syndrome has become a major public health problem worldwide and represents a common clinical condition in countries with a high incidence of obesity and western dietary patterns, strategies to control weight gain such as the appetite regulation are needed. In this study, the satiating effect of goat dairy products, traditionally part of the Mediterranean diet, was evaluated in healthy adults and compared for the first time to cow dairy products. Although the results of our study show no differences in the subjective appetite feeling between a goat dairy and a cow dairy based breakfast, except for the desire to eat rating; however, interestingly they provide evidence of a moderately higher appetite suppressor potential of goat dairy in comparison to cow dairy products. Regarding the blood parameters potentially associated with the mechanisms by which dairy products might be affecting satiety and energy intake [21,23,50,51], GLP-1 levels were associated with the appetite ratings “Hunger” and “Desire to eat” after G-Breakfast, while triglyceride levels were associated with the combined satiety index after the C-Breakfast. Additionally, changes in all the appetite ratings were independent of plasma glucose or insulin levels.

## Figures and Tables

**Table 1:** Total macronutrient composition and energy content.

	Cheese (40 g)		Milk (200 g)		Breakfast	
	Goat	Cow	Goat	Cow	Goat	Cow
Energy (kcal)	133.2	150.4	90.0	86.8	332.00	346.00
Total protein (g)	8.14	11.6	6.60	6.0	17.26	20.72
Total fat (g)	11.08	11.48	3.20	3.2	14.68	15.08
Total carbohydrates (g)	0.20	0.20	9.00	8.8	32.40	32.2
Dietary fiber (g)	-	-	-	-	0.88	0.88

**Table 2:** Baseline characteristics at test day.

	G-Breakfast	C-Breakfast
Glucose (mg/dL)	77.36 ± 16.02	80.88 ± 12.73
Insulin (mU/mL)	9.02 ± 6.04	11.69 ± 7.19
Cholesterol (mg/dL)	175.27 ± 28.34	172.12 ± 25.56
HDL-Cholesterol (mg/dL)	62.42 ± 17.60	61.88 ± 17.60
Triglycerides (mg/dL)	90.88 ± 58.22	91.27 ± 29.79
Free fatty acids (mg/dL)	0.54 ± 0.21	0.49 ± 0.24
